# Curcumin Alters Neural Plasticity and Viability of Intact Hippocampal Circuits and Attenuates Behavioral Despair and COX-2 Expression in Chronically Stressed Rats

**DOI:** 10.1155/2017/6280925

**Published:** 2017-01-11

**Authors:** Ga-Young Choi, Hyun-Bum Kim, Eun-Sang Hwang, Seok Lee, Min-Ji Kim, Ji-Young Choi, Sung-Ok Lee, Sang-Seong Kim, Ji-Ho Park

**Affiliations:** ^1^Department of East-West Medicine, Graduate School of East-West Medical Science, Kyung Hee University, Deogyeong-daero, Giheung-gu, Yongin 446-701, Republic of Korea; ^2^Department of Pharmacology, Perelman School of Medicine, University of Pennsylvania, Philadelphia, PA 19104, USA; ^3^Department of East-West Medical Science, Graduate School of East-West Medical Science, Kyung Hee University, Deogyeong-daero, Giheung-gu, Yongin 446-701, Republic of Korea; ^4^Department of Oriental Medicinal Materials and Processing, College of Life Science, Kyung Hee University, Deogyeong-daero, Giheung-gu, Yongin-si, Gyeonggi-do 446-701, Republic of Korea; ^5^Department of Pharmacy, Hanyang University, Hanyangdaehak-ro, Sangnok-gu, Ansan, Gyeonggi-do 15588, Republic of Korea

## Abstract

Curcumin is a major diarylheptanoid component of* Curcuma longa* with traditional usage for anxiety and depression. It has been known for the anti-inflammatory, antistress, and neurotropic effects. Here we examined curcumin effect in neural plasticity and cell viability. 60-channel multielectrode array was applied on organotypic hippocampal slice cultures (OHSCs) to monitor the effect of 10 *μ*M curcumin in long-term depression (LTD) through low-frequency stimulation (LFS) to the Schaffer collaterals and commissural pathways. Cell viability was assayed by propidium iodide uptake test in OHSCs. In addition, the influence of oral curcumin administration on rat behavior was assessed with the forced swim test (FST). Finally, protein expression levels of brain-derived neurotrophic factor (BDNF) and cyclooxygenase-2 (COX-2) were measured by Western blot in chronically stressed rats. Our results demonstrated that 10 *μ*M curcumin attenuated LTD and reduced cell death. It also recovered the behavior immobility of FST, rescued the attenuated BDNF expression, and inhibited the enhancement of COX-2 expression in stressed animals. These findings indicate that curcumin can enhance postsynaptic electrical reactivity and cell viability in intact neural circuits with antidepressant-like effects, possibly through the upregulation of BDNF and reduction of inflammatory factors in the brain.

## 1. Introduction

Excessive stress can cause anxiety, tension, and depression and have an adverse effect on normal life. Chronic stress can alter nervous, endocrine, and immune systems, thus affecting the overall homeostatic mechanisms in the body [1]. Stress influences have been widely recognized and studied in numerous research projects [2, 3]. Depression under stressful situations evokes a variety of cognitive symptoms, as well as physical changes such as lack of motivation, leading to chronic melancholy. A recent brain imaging study revealed changes in neurotransmitters in the brains of patients under depression, implying an etiology associated with depression [4].

Continuous progress has been achieved in the pursuit of effective antidepressants. Various classes of antidepressants have been developed based on the neurotransmitter system and used in clinical settings, including selective serotonin reuptake inhibitor (SSRI), norepinephrine-dopamine reuptake inhibitor (NDRI), and serotonin-norepinephrine reuptake inhibitor (SNRI). However, these antidepressants demonstrate gradual effects with slow onset and take least 4 to 6 weeks to achieve their effect. Also, there are issues of side effects including dry mouth, constipation and orthostatic hypotension, which have been frequently reported during the early phase of the treatment period. Therefore, there is a high demand for antidepressants with fewer side effects [5].

Recently, tension relievers such as L-theanine and anxiolytics such as alprazolam (Xanax, Pfizer Inc.) have been used to alleviate stress [6]; however, these drugs cannot become standard treatments because of their significant side effects. Therefore, botanical extracts are under investigation as alternatives for relieving stress since they are considered to have fewer side effects than chemical drugs.

Curcumin is a major diarylheptanoid and polyphenol component of* Curcuma longa* with both medical and nutritional values. It is extracted from the dry rhizome of* Curcuma longa* Linn (Zingiberaceae), a perennial herb that is widely cultivated in tropical regions of Asia [7]. It has been used for centuries in indigenous medicine for the treatment of a variety of inflammatory conditions and other diseases. Curcumin was also shown to significantly reverse chronic stress-induced behavioral and cognitive alterations in stressed rats [8, 9].

Previous research findings demonstrated that BDNF and COX-2 play an important role in the pathogenesis of depression and inflammation. Curcumin has already been shown to stimulate BDNF and inhibit COX-2 in chronically stressed rat models; however, the neuroprotective effect of curcumin in the chronically stressed rat model remains unknown. Therefore, we investigated the function of curcumin in modulating depressant-like behaviors in chronically stressed rats. This study reveals the neuroprotective effects of curcumin associated with antidepression activity through long-term depression- (LTD-) associated neural plasticity.

## 2. Materials and Methods

### 2.1. Materials

Curcumin, dimethyl sulfoxide (DMSO), lipopolysaccharides from* Escherichia coli* (L2637), HEPES (H4034), L-glutamine (G-8540), D-glucose (G-7528), and kainic acid were purchased from Sigma (St. Louis, MO, USA). Minimum essential medium (MEM, LM 007-01), Hank's balanced salt solution (HBSS, LB 003-01), and horse serum (S 104-01) were purchased from JBI (Daegu, South Korea). Penicillin streptomycin was obtained from Gibco BRL (LS 202–02, USA).

### 2.2. Animal Models

Sixteen 6-week-old male Sprague-Dawley (SD) rats with an average weight of 71 ± 8 g were used for in vivo experiments. SD rats were purchased from Orient Bio Inc. (Gyeonggi, Republic of Korea) and were fed on a standard pellet diet supplied by Orient Bio Inc. They were housed two per cage at the animal facility of the Kyung Hee University under maintained conditions of temperature 26 ± 1°C and relative humidity 60 ± 5% with a 12 h light/dark cycle (lights on 8:00 a.m., lights off 8:00 p.m.). All rats were allowed to acclimatize to the laboratory conditions for at least 1 week prior to the experiments. They were given tap water and a standard diet ad libitum during the experiments except when the chronic stress procedure required deprivation.

### 2.3. Drug Administration and Experimental Groups

Curcumin was dissolved in DMSO at a concentration of 10 mg/mL and the concentration of DMSO did not exceed 0.1% of the total volume.

**Figure 1:**
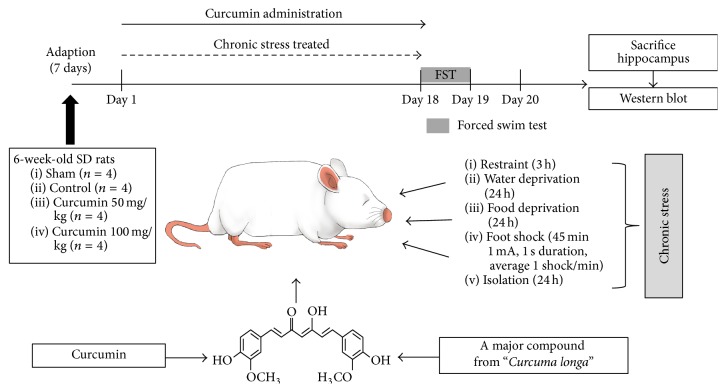
Experimental schedules for curcumin administration to produce an antidepressant effect in chronic stress-induced rats. The experiment was designed to explore the efficacy of curcumin against chronic stress-induced depression in an animal model using behavioral and biological methodologies. All animals were given distilled water for one week after arrival to habituate them to laboratory conditions. Starting on the first day, sham (*n* = 4) SD rats and control (*n* = 4) SD rats were fed 10 mL/kg distilled water until the end of the experiment. For the treatment groups, curcumin 50 (*n* = 4) SD rats were fed 50 mg/kg curcumin and curcumin 100 (*n* = 4) SD rats were fed 100 mg/kg curcumin. Curcumin was dissolved in DMSO. Stress was administered once a day over a period of 18 days in the control and curcumin groups. Behavioral testing commenced 60 min after the last curcumin treatment. On the last day, the rats were killed for biological experiments.

Sixteen rats at the age of 6 weeks were randomly assigned to four groups (*n* = 4/group): Group 1 received 0.1% DMSO (10 mL/kg) and served as the sham; Group 2 was exposed to chronic stress and received 0.1% DMSO (10 mL/kg) as the control; Group 3 was exposed to chronic stress and received curcumin at a concentration of 50 mg/kg; Group 4 was exposed to chronic stress and received curcumin at a concentration of 100 mg/kg. The curcumin was injected at a dose of 1 mL per 100 g body weight. All treatments consisted of an oral injection between 10:00 and 11:00 a.m. administered once a day for 18 consecutive days. All the animals under test had been survived throughout the procedures, which were analyzed for statistical difference using ANOVA. All animal procedures complied with the Institutional Care and Use Committee (KHUASP(SE)-15-024) of Kyung Hee University and were performed in accordance with the guiding principles for the care and use of animals approved by the Council of the National Institutes of Health Guide for the Care and Use of Laboratory Animals (Figure 1).

### 2.4. Chronic Stress Procedure

The procedure of chronic stress induction was conducted as previously described [10], with slight modifications. Stress was administered once a day over a period of 18 days between 2:00 p.m. and 5:00 p.m. The chronic stress protocol consisted of a variety of stressors: restraint (3 h), water deprivation (24 h), food deprivation (24 h), foot shock for 45 min (1 mA, 1 s duration, average 1 shock/min), or isolation (24 h). These stressors were randomly scheduled for 18 days. The control group was housed in a separate room without contact with the stressed group (Figure 1).

### 2.5. Forced Swim Test

The forced swim test (FST) was performed to measure behavioral despair of the rats. The test was performed as described previously with minor modifications [11]. The forced swim test was performed on the 18th day of curcumin administration. In each trial two rats were placed in two acrylic cylinders (height: 49.7 cm, diameter: 24.4 cm) containing water at 23°C with a depth of 30 cm. There was a white barrier between the cylinders to induce visual isolation. Rats were allowed a 15-minute preswim as an induction procedure. After the preswim, the animals were wiped with a towel and returned to their home cages. The cylinders were cleaned after each preswim trial with 75% alcohol to remove olfactory cues and refilled with fresh water. After 24 h the rats were put in a cylinder again for a 6 min forced swim test with a recording camera. After 6 min of swimming, the cylinder was cleaned and the water was replaced. Immobility was defined as the total time that the animal remained afloat without moving its limbs. Rodents adopt immobile positions when they lose hope of exiting the cylinder. In this circumstance, behavioral despair was defined as the immobility of a rat that has ceased any effort to escape the cylinder. Immobility was measured with a multichannel stopwatch program.

### 2.6. Tissue Preparation

After 3 weeks of treatment the rats were sacrificed. On the last day of the experiment, all rats were deprived of food or water for 24 h. The next day, the rats were anesthetized with isoflurane and blood samples from the right ventricle were collected by cardiac puncture. These samples were centrifuged at 2500 ×g for 20 min at 4°C to obtain plasma, which was divided into aliquots and stored at −80°C. The brains were rapidly excised and dissected. Tissues were stored at −80°C for later Western blot analysis.

### 2.7. Body Weight

Rats were weighed weekly from day 1 to day 20 during the procedure.

### 2.8. Western Blot Analysis

Tissues from animals in sham, control, curcumin 50 mg/kg, and curcumin 100 mg/kg groups were analyzed by Western blotting. Briefly, previously sectioned brains were removed from −80°C storage and a region of the hippocampus was dissected out on dry ice. The dissected hippocampus was homogenized by sonication in cold cell lysis buffer containing phosphatase inhibitors and a complete protease inhibitor cocktail. Hippocampus extracts were then incubated on ice for 30 min and centrifuged at 14,000 ×g for 10 min at 4°C. Protein concentrations of supernatants were measured by the Bradford protein assay [12] and equal amounts of protein were separated on a 10% SDS-PAGE gel and transferred to PVDF membranes. The membranes were blocked in TBS with 0.1% Tween 20 containing 5% dry skim milk for 1 h and incubated in 5% skim milk with primary antibodies overnight at 4°C. Antibodies used were polyclonal antibody against brain-derived neurotrophic factor (sc-33904, Santa Cruz Biotechnology), polyclonal antibody against cyclooxygenase-2 (sc-1747, Santa Cruz Biotechnology), and mouse monoclonal antibody against beta actin (sc-47778, Santa Cruz Biotechnology). Membranes were washed and further incubated for 1 h at room temperature with secondary antibodies (goat anti-mouse and donkey anti-goat IgG conjugated to horseradish peroxidase; sc-2005, sc-2056, Santa Cruz Biotechnology). After a final wash the bands were developed using a horseradish peroxidase-conjugated secondary antibody and visualized with an ECL Western Blotting Detection System (ATTO system). All experiments were repeated at least three times with different batches of tissue samples and the results were fully reproducible.

### 2.9. Organotypic Hippocampal Cultures

Organotypic hippocampal slice cultures (OHSCs) were prepared as previously reported [13]. All procedures were carried out in a sterile environment. In brief, 7-day-old Sprague-Dawley rats were decapitated and their brains were quickly removed. The brains were immediately immersed in ice-cold HBSS medium (LB 003-01, Sigma) with 20 mM HEPES (H-4034, Sigma). The frontal cortex and the cerebellum were delicately removed. The hippocampus was harvested and sectioned transversely at 350 *μ*m using a tissue chopper (Mickle Laboratory Engineering Co., Surrey, UK). Four to five slices were plated on each 0.4 *μ*m culture insert (Millicell-CM; Millipore, Bedford, MA, USA), which were set into wells of a 6-well plate filled with 1 mL of culture medium (MEM, supplemented with 20 mM HEPES, 25% (v/v) Hank's balanced salt solution, 6 g/L D-glucose, 1 mM L-glutamine, 25% (v/v) heat-inactivated horse serum, and 1% penicillin streptomycin, pH 7.1). The culture slices were maintained at 36°C in a 5% CO_2_ and 95% O_2_ humidified incubator. The culture medium was changed three times a week and the sections were cultured for 14 days before experimental treatments.

### 2.10. Measurement of Propidium Iodide (PI) Uptake

Cell death was assessed by fluorescent image analysis of propidium iodide (PI; Sigma) staining. At 2 weeks after OHSC, the PI stock (5 *μ*g/mL) was added to serum-free culture medium and the slices were incubated in the dark at 36°C. After 2 h incubation, the tissues were washed with culture medium and cell death in the hippocampal layers was detected by lack of PI uptake.

Curcumin was dissolved in DMSO and stored at −20°C until use. Aliquots were diluted in culture media. OHSCs were treated with 5 *μ*M kainic acid (KA) and 10 *μ*M curcumin [14]. The effects of KA and curcumin treatment for 24 and 48 h on cell death were observed by PI staining.

PI stained images were captured using a laser scanning microscope (LSM510, Carl Zeiss, Mannheim, Germany). Areas of PI uptake were measured with LSM 510 software (Carl Zeiss) and the image intensity was analyzed using the Image J program.

### 2.11. Preparation of Organotypic Hippocampal Slice Tissue on Microelectrode Array Probes

A single stabilized hippocampal slice was carefully removed from a membrane insert with a needle and placed on an 8 × 8 microelectrode array (MEA) of 10 *μ*m diameter electrodes spaced 100 *μ*m apart (Multi Channel Systems, Reutlingen, Germany) that was precoated with 0.01% polyethylenimine. MEAs consist of a high-density electrode array with stimulator, amplifier, temperature control unit, and computer for data acquisition. The slice was stabilized in artificial cerebrospinal fluid (aCSF: 114 mM NaCl, 26 mM NaHCO_3_, 10 mM glucose, 3 mM KCl, 2 mM CaCl_2_, 1 mM MgCl_2_, 20 mM HEPES, pH 7.4) for 30 min at 33°C with 95% O_2_ and 5% CO_2_ gas aeration. Extraneous aCSF was then removed using a pipette. The MEA containing the hippocampal slice was transferred to an MEA1060 amplifier interface. The solution in the array was grounded using an Ag/AgCl pellet. Data were sampled from each channel at a speed of 25 kHz and recorded using Recorder-Rack software (MEA systems, MCS software). The stimulating channel was disconnected from the sampling device during stimulation. After the experiment, the array was cleaned with 2% ultrasonol 7 (Carl Roth GmbH, Karlsruhe, Germany) in distilled water for approximately 30 min, rinsed with distilled water, and then kept in distilled water at room temperature.

### 2.12. Induction of LTD in Hippocampal Slices

**Figure 2:**
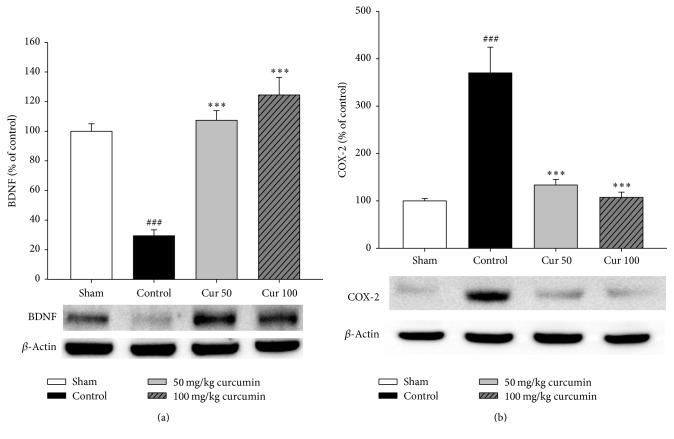
Effect of curcumin on the protein expression of brain-derived neurotrophic factor (BDNF) and cyclooxygenase-2 (COX-2) in rats with chronic stress-induced hippocampal impairment (*n* = 4/group). (a) The protein level of BDNF in rat hippocampus was measured by Western blot analysis using anti-BDNF specific antibody. (b) The protein level of COX-2 in rat hippocampus was measured by Western blot analysis using anti-COX-2 specific antibody. The data were normalized against *β*-actin levels and expressed as percentage of control values. ^###^*p* < 0.001 versus sham group. ^*∗∗∗*^*p* < 0.001 versus control group.

The MEA system is composed of a 64-channel array with four stimulating and 60 recording electrodes (STG1004; Multi Channel Systems GmbH, Germany), an amplifier (MEA1060; Multi Channel Systems GmbH, Germany), temperature control units (Multi Channel Systems GmbH, Germany), and data acquisition software (http://www.multichannelsystems.com/) [15]. The amplifier was placed in a Faraday cage. Bipolar electrical stimulation was applied to the CA2 stratum radiatum region to stimulate the Schaffer collateral (SC) and commissural pathways. The intensity of bipolar test pulse stimulation was set at 100 mA; this value was optimized to provide 40–65% of the maximum tissue response and was delivered once every 60 sec. Baseline responses were evoked for at least 30 min, of which the last 40 minutes were recorded, before application of the low-frequency stimulation (1 Hz for 15 minutes; 900 total pulses; Figure 2(a)) to induce LTD. After the conditioning stimulation, field excitatory postsynaptic potentials (fEPSPs) were recorded every 60 sec for another 50 min from 59 microelectrodes spanning the hippocampus. During experiments the slices were continuously perfused with fresh aCSF solution or aCSF with drugs (dissolved with 95% O_2_, 5% CO_2_) at a rate of 3 mL/min and carbogen consumption of 161/h.

### 2.13. Electrophysiology Data Processing

MC_Rack (v.3.2.1.0, Multi Channel Systems) was used to digitize the analog MEA signal and isolate EPSPs from triggering amplitudes greater than 40 mV, and a custom MATLAB (v.7.0.1, Mathworks, Inc.) program was used to remove stimulus artifacts and integrate the evoked field potential trajectory, as reported previously [15, 16].

### 2.14. Statistical Analysis

The results in the figures are expressed as the mean ± standard error of the mean (SEM). Statistical comparisons were conducted using one-way and two-way analysis of variance (ANOVA) followed by Duncan's post hoc multiple comparison test using SPSS 20.0 for windows (SPSS Inc., Chicago, IL, USA). Differences were considered statistically significant if the *p* value was less than 0.05.

## 3. Results

### 3.1. Effects of Curcumin on Body Weight

Body weight measurements are shown in Figure 5. Before the experiment, all rats had an average body weight of 71 ± 2 g. At the end of 3 weeks, the body weight of sham and control groups were different; the sham group showed a significant increase in body weight whereas the control stressed group did not. The body weight of rats in the sham group increased by 85 ± 7.4 g compared to 66 ± 4.9 g for control rats. A valid difference was not detected with GLMM or Kruskal-Wallis test.

### 3.2. Effects of Curcumin on Forced Swim Test

The FST was applied on day 18. As shown in Figure 6, statistical analysis of the FST data showed differences in the immobility time among the four groups. The chronically stressed control group spent a longer time immobile than the sham group; the immobility time of the control group was 15.9% higher than that of the sham group. Treatment with 50 mg/kg curcumin decreased the immobility time by 8.6% compared to the control group. Moreover, treatment with 100 mg/kg curcumin significantly reduced immobility time by 48.8% compared to the control group (*p* < 0.05). These results showed that curcumin has antidepressant effects in the FST as evidenced by decreased immobility.

### 3.3. Effects of Curcumin on BDNF Expression

The Western blot results demonstrated that BDNF protein levels were decreased by 70.6% in the hippocampus of the stressed control group (*p* < 0.001 versus nonstressed sham group, Figure 2). However, treatment with curcumin significantly increased the BDNF protein levels in the hippocampus of stressed rats compared to control (*p* < 0.001, Figure 2). Specifically, curcumin at doses of 50 and 100 mg/kg (p.o.) increased BDNF protein levels by 78.0% and 95.1%, respectively, compared to the control group. These results indicate that curcumin enhanced the BDNF level in hippocampus of chronic stressed rats. Treatment with several doses of curcumin treatment showed that 100 mg/kg (p.o.) was most effective. However, both doses of curcumin tested had a significant effect on the expression ratio of BDNF/*β*-actin.

### 3.4. Effects of Curcumin on COX-2 Expression

We also measured the expression level of the inflammation maker COX-2 by Western blot analysis. COX-2 protein levels were significantly increased in the hippocampus of the stressed group (*p* < 0.001 versus nonstressed sham group). As shown in Figure 2, COX-2 levels after treatment with 50 mg/kg and 100 mg/kg curcumin treatment were reduced by 236.6% and 262.7%, respectively, compared to the control group (*p* < 0.001). These results indicate that curcumin decreased the COX-2 level in the hippocampus of chronic stressed rats. Both doses of curcumin had a significant effect on COX-2 expression, but curcumin 100 mg/kg was so effective that it showed no significant difference from the sham group.

### 3.5. Effects of Curcumin on Neuronal Cell Death

**Figure 3:**
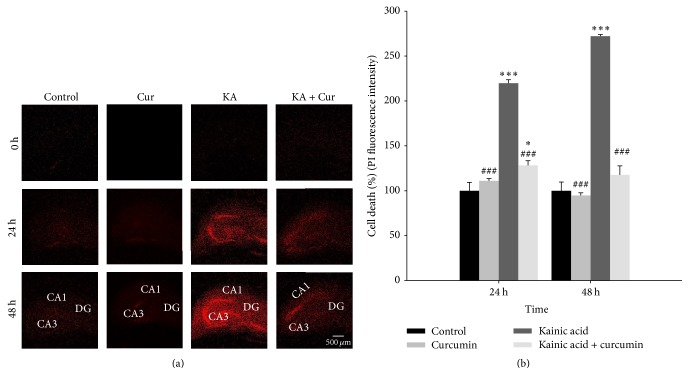
Detection of neuronal cell death in organotypic hippocampal slice cultures (OHSCs) with kainic acid (KA) and curcumin treatment by propidium iodide (PI) staining. OHSCs were treated with 10 *μ*M curcumin and 5 *μ*M KA. (a) Representative images of PI uptake in OHSCs. Red represents PI fluorescence, which indicates cell membrane damage. Scale bar = 500 *μ*m. (b) Quantification of hippocampal cell death. Data are shown as the percentage cell death. The fluorescence intensity of the control group was designated as 100%. Differences in PI fluorescence intensities among control, control + curcumin only, KA-only-treated group, and KA with curcumin groups were observed by fluorescence microscopy. The PI value is shown as mean ± SEM. Four hippocampal slices were used in each group. ^*∗*^*p* < 0.05, ^*∗∗∗*^*p* < 0.001 versus control group. ^###^*p* < 0.001 versus the KA-only-treated group.

Average PI update in each group was measured after 2 days of treatment with KA alone and KA with curcumin. As shown in Figure 3(b), 5 *μ*M KA caused neuronal cell death in the hippocampus at 24 and 48 hours after application. When the value of the control group was set as 100% cell death, the death rate of slices incubated with KA (5 *μ*M) with curcumin (10 *μ*M) was 128.3% (24 h) and 117.7% (48 h). Treatment with 10 *μ*M curcumin significantly reduced the PI fluorescence (Figure 3). Therefore, curcumin treatment counteracted the effect of KA resulting in a similar death rate between the sham and KA + curcumin groups.

### 3.6. The Effects of Curcumin on LTD in Hippocampal Tissue

**Figure 4:**
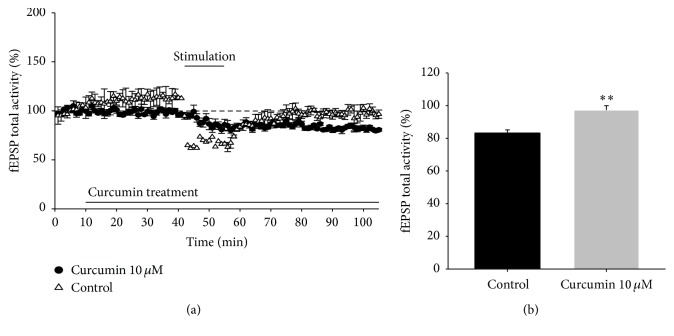
Effects of curcumin on long-term depression (LTD) in rat hippocampal tissue (*n* = 3-4/group). (a) Time course of LTD from all recordings made from control or curcumin-treated (10 *μ*M) hippocampal tissue; (b) average LTD amplitude measured 30–40 min after LFS; ^*∗∗*^*p* < 0.01 versus the control group.

LTD was recorded over 100 min and the average of fEPSPs was analyzed when applying LFS for approximately 30–40 min. According to the total activity of fEPSP calculated from the experimental results, slices perfused with aCSF containing curcumin showed an increase in LTD (Figure 4). In control slices from the rat hippocampus, fEPSP was potentiated to 83.25 ± 1.92% (*n* = 3-4). In curcumin (10 *μ*M)-treated slices the fEPSP was potentiated to 97.25 ± 2.69% (*n* = 3-4, *p* < 0.05 versus control).

## 4. Discussion

**Figure 5:**
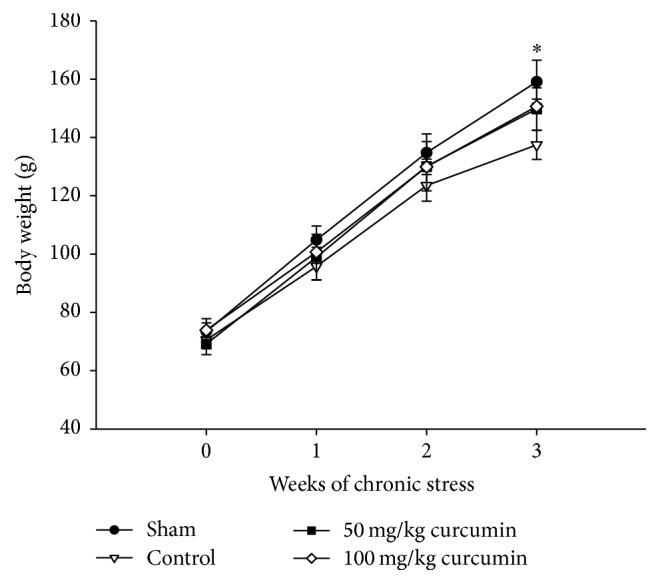
Effects of chronic stress and curcumin treatment on body weight in SD rats. Data represent mean ± SEM (*n* = 4/group). ^*∗*^*p* < 0.05 by GLMM or Kruskal-Wallis test.

**Figure 6:**
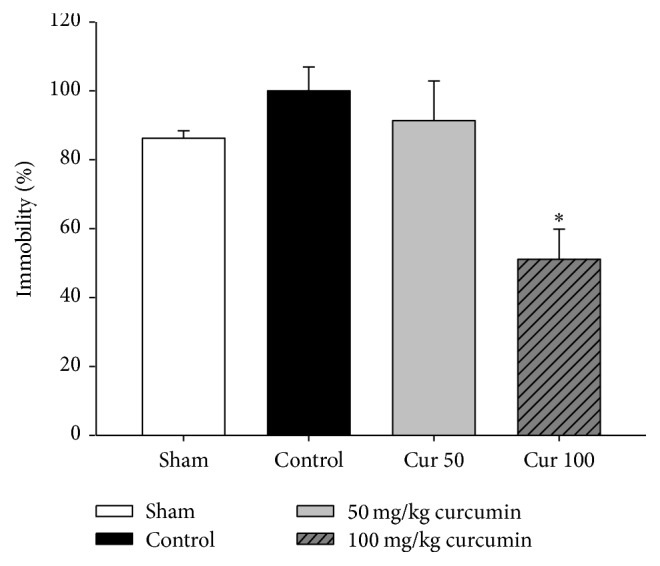
Effects of curcumin administration on behavior immobility in forced swim test. Curcumin was administered for 18 days before the FST. The immobility time was measured during a 6 min experimental session. Data are shown as mean ± SEM. According to Kurtosis figure or Kurtosis/Kurtosis error, each group had normal distribution. ^*∗*^*p* < 0.05 versus control by ANOVA. In addition, ^*∗*^*p* < 0.05 by Kruskal-Wallis test.

The effect of curcumin on neural plasticity and cell viability has been investigated in in vitro and in vivo experimental models. There have been controversies concerning the effect of stress on body weight [17, 18]: one study reported that body weight was decreased in the stressed animal model [18], whereas another suggested that there was no significant difference in body weight in the stressed model compared to the placebo [17, 19]. In this experiment, the control group showed a decrease in body weight compared to the sham group that was not affected by stress (Figure 5). These contradictory findings between studies could be due to differences in the stressor used or the level of stress.

The antidepressant activity of curcumin was previously evaluated in forced swim tests using chronic stress-induced rat models [20]. Oral treatment with 50 and 100 mg/kg/day curcumin for 18 days showed an 8.6% and 48.8% reduction in immobility, respectively, compared to the control group. Curcumin-treated groups showed reduced immobility in a dose-dependent manner, indicating that curcumin may possess antidepressant properties. BDNF is a major neurotrophic factor that plays an important role in the maintenance and the survival of neurons [21–23]. Upon binding to TrkB receptor, BDNF induces glutamate release from presynaptic region following MAPK/ERK and CaMKII signaling activation in postsynaptic neuron. It results in elevation of neural plasticity [2]. The curcumin-treated group showed an increase in BDNF protein expression in the hippocampus region suggesting a strong link between antidepressant effects and BDNF expression. This also suggests that curcumin might partially exert its antidepressant activities by attenuating or reversing abnormalities in BDNF expression induced by chronic stress [24] (Figure 2).

The neuroplastic changes in depression can be mediated through neuroimmune processes, including humoral and cellular neuroimmunological factors. Inflammatory cytokines such as interleukin-1*β* (IL-1*β*) and tumor necrosis factor (TNF) increase COX-2 protein expression in various human cell types [25]. COX-2 is an enzyme to covert arachidonic acid into prostaglandins such as PGD, PGI2, and PGE2 in response to neuroinflammatory factors [1]. Therefore, its expression level is widely used as an indicator to represent degree of inflammation. In line with it, COX-2 inhibitors have demonstrated neuroprotective effects in numerous CNS-related disorders [26]. Curcumin has also shown antidepressant-like effects in a LPS-induced depression model, which could be attributed to reduced levels of proinflammatory cytokines like iNOS and COX-2 via the NF-*κ*B signaling pathway. Our results imply that immobilization by stress could cause enhanced COX-2 protein expression in the cortex, particularly the hippocampus regions [27] (Figure 2). In the present study, curcumin reduced COX-2 protein expression in the hippocampus of stress-induced rats. These findings suggest that curcumin partially induces antidepressant activities by attenuating or reversing chronic stress-induced abnormalities resulting from COX-2 protein expression. Likewise, curcumin pretreated rat models showed a reduced NO level under KA treatment, implicating a protective effect of curcumin against KA-induced neuronal loss [28]. It has been reported that seizure induced by KA results in hippocampal cell death and upregulation of caspase-3, GFAP, eNOS, and HO-1. In contrast, curcumin prevented neuronal cell death and attenuated the upregulation of these proteins in astrocytes [29].

Currently, the effects of curcumin on LTD are not fully understood. This study demonstrated the neuroprotective effects of curcumin. Long-term potentiation (LTP) is defined as a strong depolarization that occurs when the excitatory neurotransmitter glutamate in presynaptic terminals is recognized by the postsynaptic glutamate receptor (NMDA receptor). LTP increases the synaptic efficacy in the mammalian brain and is also associated with learning and memory [30]. However, storage of information requires mechanisms for weakening, as well as strengthening. LTD is the functional inverse of LTP and a candidate mechanism for learning and memory development in the hippocampus [31]. We demonstrated that hippocampal field potential LTD was prevented by curcumin. The results of this study might advance our understanding of the neuroprotective effects of curcumin for memory storage and restoration (Figure 4).

In this study we demonstrated the anti-inflammatory and antidepressant properties of curcumin. We also observed the neuroprotective effects of curcumin in a LTD model. Based on these findings, we believe that curcumin might be a potential candidate for anxiolytic or antidepressant therapy.
